# Personal, Social and Environmental Risk Factors of Problematic Gambling Among High School Adolescents in Addis Ababa, Ethiopia

**DOI:** 10.1007/s10899-013-9410-9

**Published:** 2013-09-29

**Authors:** Tariku A. Abdi, Robert A. C. Ruiter, Tamirie A. Adal

**Affiliations:** 1 Ethiopia Field Office, Johns Hopkins University Center for Communication Programs (JHU CCP), P.O. Box 1865, 1250 Addis Ababa, Ethiopia; 2 Department of Work and Social Psychology, Maastricht University, Maastricht, The Netherlands; 3 School of Psychology, Addis Ababa University, Addis Ababa, Ethiopia

**Keywords:** Risk factors, Gambling, Problematic gambling

## Abstract

Understanding risk factors of problematic gambling is prerequisite to effective intervention design to alleviate the negative consequences of gambling. This study explored the personal, social and environmental risk factors of problematic gambling in four high schools in Addis Ababa, Ethiopia, among students (N = 422) ranging from 12 to 21 years of age. Results from the cross-sectional survey showed that personal feelings (e.g., self-esteem, false perceptions about winning, drug abuse), social factors (e.g., peer influence, parental gambling), and environmental factors (e.g., accessibility of gambling venues, advertisements) were significant correlates of problematic gambling. The study also revealed that men were more at risk for severe problematic gambling than females. Among the identified types of gambling activities, the most prevalent ones were playing cards followed by flipping coin and pool gambling while internet gambling was among the least reported gambling activities. By identifying personal, social and environmental correlates of risky gambling activities this study provides evidence-based information for the systematic design and evaluation of educational interventions to prevent problematic gambling in young people.

## Introduction

With the expansion of gambling venues, including the Internet, adolescents are widely engaging in gambling activities. Studies indicate that gambling is increasingly becoming one of the most popular leisure activities among adolescents worldwide, with rates of problematic gambling being higher in adolescents than in adults (Derevensky et al. 2004; Dickson and Derevensky 2006; Winters et al. 2002). While gambling is widely accepted as a form of entertainment, problematic gambling remains a social and public health issue. Although problematic gambling affects only a small minority of individuals, the negative consequences are usually widespread with serious psychological, social, and economic implications not only for the individual gambler, but also bearing severe personal and economic costs for family members and the wider community and society (e.g., Derevensky et al. 2004; Ellenbogen et al. 2007; Lesieur 1998; Shaffer and Korn 2002). In adolescents, problematic gambling has been shown to result in increased delinquency and crime, the disruption of relationships with family and peers, and the impairment of academic performance and work activities (Derevensky and Gupta 1998). Furthermore, if left unchecked, frequent gambling in adolescence may develop into problematic gambling in adulthood (Carlson and Moore 1998). It is therefore of crucial importance to understand adolescent gambling, not only to reduce negative personal and social consequences associated with gambling, but also to arrest the development of problematic gambling before it transfers to adulthood (Carlson and Moore 1998).

Problematic gambling is defined in many texts in statements of interrelated variables. The term problematic gambling is used by lay and professional audiences to indicate the patterns of gambling behavior that compromises, disrupts or damages personal, family or vocational pursuits (Volberg and Stuefen 1994). Similarly, clinical psychologists have defined problematic gambling not only in terms of the frequency and intensity of engagement and the severity of negative outcomes as reflected for instance in the amount of debts as a result of engagement in gambling, but also in the extent to which gambling has a disruptive impact on daily life referring closely to the addictive component of gambling (Shehu 2004).

There are various international instruments to diagnosis problematic gambling. Among the widely used instruments, the Diagnostic and Statistical Manual of Mental Disorders Fourth-Version Text Revision (DSM-IV-TR) developed by the American Psychiatrists Association (APA, 2000, p. 674) categorizes gambling using 10 criteria: preoccupation, tolerance, withdrawal, loss of control, escape (gambling to escape from problems or gambling when bad feeling), chasing, lying to family and therapist about the problems of gambling, illegal acts, and relational disruption. Among these criteria, those who score positively on four or more criteria are said to be problematic or pathological gamblers. Those who score 2–3 positively are called at risk for severe problematic gambling, and finally gamblers 0–1 positive scores are called social/non-problematic gamblers. Other instruments like the Diagnostic and Statistical Manual of Mental Disorders Fourth-Version Revised for Juveniles (DSM-IV-J; Derevensky and Gupta 2000) and Gamblers Anonymous Twenty Questions (GA-20) which is developed by Gamblers Anonymous (Derevensky and Gupta 2000) diagnosed problematic gambling in terms of personal, social and economic costs. In adolescents, problematic gambling may be viewed as a gambling activity that may interfere with daily activities as reflected in cutting classes, relationship disruption/problem, and gambling addiction.

### Risk Factors and Correlates of Problematic Gambling

Problematic gambling is governed by a complex set of interrelated factors, causes and determinants ranging from biology and family history to social norms and existing statutes (Messerlia et al. 2005) encompassing ecological, psycho-physiological, developmental, cognitive, and behavioral components (Derevensky et al. 2004).

Among the personal (psychological) risk factors, Tang and Oei (2011) reported that life stress was being associated with erroneous gambling cognition, while Dickson et al. (2008) in their study revealed that trait anxiety, risk propensity and ineffective coping were found to be positively correlated with gambling problems. Gillespie et al. (2007) also reported that problematic and pathological gamblers engage in gambling to escape problems, to alleviate depression, to cope with loneliness, to relax, and to interact socially with others.

At the social level, Carlson and Moore (1998) found problematic gambling to be associated with familial and community factors such as family gambling history, and lax or laissez-faire social regulation on gambling. Similarly, parental and peer modeling of gambling behavior were found to be positively related to adolescents’ participation in gambling activities (Magoon and Ingersoll 2006).

At the environmental level, advertisements on gambling and easy accessibility of gambling venues have been associated with risk factors for problematic gambling. Monagan et al. (2008) revealed in their study that 42 % of youth reported gambling advertisements make them want to try gambling and 61 % imagine or dream about what they could buy with their winnings. Furthermore, Lambos et al. (2007) revealed that problematic gamblers were significantly more likely than those ‘at risk’ and those ‘not at risk’ to report having watched TV-poker games, to have enjoyed watching the programs, and to have been being encouraged by the programs to play card games for money. Hing and Nisbet (2010) in their qualitative study of physical, social and cognitive accessibility of gambling venues found out that there exists a possible relationship between heightened accessibility to gambling and the development and maintenance of problematic gambling amongst employees at gambling venues.

Studies also indicate that problematic gambling is positively correlated with substance/drug abuse. Delfabbro et al. (2005) in their study of adolescent gambling of ages 7–12 years found out that three quarters of problematic gamblers reported drinking alcohol on a weekly basis compared with half of the non-problematic sample. The prevalence of smoking by problematic gamblers was four times the rate recorded for non-problematic gamblers, for marijuana over six times the rate, and for harder drugs, 10–20 times the rate recorded for the non-problematic sample.

#### The Present Study

There is lack of evidence on risk factors for problematic gambling among certain cultural groups. Most studies on adolescent gambling are conducted in the context of Western cultures. Ellenbogen et al. (2007) in their cross-cultural study of gambling behaviors among adolescents of Anglophone (English), Francophone (French), and Allophone (other) in Quebec found out that cultural factors influence gambling behaviors and problematic gambling rates. The results of their study indicated that some meaningful between-group differences were found with respect to factors related to problematic gambling (i.e., comorbidity with other risk factors, coping, family functioning and resiliency).

Problematic gambling is hardly systematically studied in the African context except for South Africa. According to a report of Nzimande et al. (2010), the national prevalence of problematic gambling in South Africa is estimated at 3 %, whereas Wiebe and Volberg (2007) in their overview of gambling literatures indicated that the prevalence of problematic gambling in South Africa fluctuated between 4.2 and 6.8 % from 2001 to 2005. Rule and Sibanyoni (2000) reported that the legalization of the gambling industry in South Africa was associated with increases in societal problems among which prostitution, theft, rape, robbery, and assault.

With respect to the context of Ethiopia, gambling is not technically illegal. In the streets of the capital city Addis Ababa, there are plenty of gambling activities played for money with state-owned lotteries, playstations, pool houses, and table football being the most prevalent ones. The National Lottery Administration (NLA) Ethiopia has the role of regulating and controlling the activities related to gambling (National Lottery Administration (NLA), Ethiopia, 1961). In 1980s, a casino did exist at the Ghion Hotel in Addis Ababa, Ethiopia, but after a brawl which resulted in the death of a patron, it was shut down by order of the then Ethiopian prime minister. In 1995 and 2006, two project proposals for a new casino were presented to parliament, but both were not successful (retrieved from http://onlinecasinosuite.com/gambling/ethiopia/).To date, there is no scientific research conducted on gambling in Ethiopian context. Therefore, it is imperative to have cultural specific research findings on gambling in order to deal with the risk factors and other psychosocial and behavioral correlates of problematic gambling.

In this study we surveyed the personal, social, and environmental risk factors of problematic gambling in Ethiopia with a specific focus on adolescents as these are most vulnerable to getting involved in gambling due to the high unemployment rates. By exploring the risk factors of gambling behavior in adolescents, this study aims to identify target variables for future educational and policy interventions to prevent problematic gambling in adolescents in Ethiopia.

## Methods

### Participants and Procedures

A cross-sectional survey study was conducted among regularly attending high school adolescents in Addis Ababa, Ethiopia in 2009. At the time, in Addis Ababa there were about 112 high schools with a total of 114,094 students (Addis Ababa in the Past and its Prospects in the New Millennium 2006/07). The researcher and first author of the current paper contacted the representatives of four high schools with a total of seven grade 9 and 10 classes with 422 students (227 males and 195 females) and got permission to conduct the study. In each of the schools, the researcher contacted classroom teachers for their willingness to cooperate in data collection by devoting a one period (50 min) class meeting. Following the permission of the schools and with the help of the teachers, the researcher approached the students. The researcher explained the objectives of the study for the students and assured them about the confidentiality of their responses. Students did not provide their names and were not asked any other personal identification. Only students who had played any game in money or betted on unknown results were eligible to fill in the questionnaires. But students who did not play any game in money or bet on unknown results were also counted in order to assess the frequency of gambling in the target population. The students were instructed to finish the questionnaire in the class within the 50 min class meeting and return the questionnaire to their classroom teachers or researcher before they left the class. Out of the 422 target population, 307 (about 73 % with males 41 % and females 32 %) reported that they have gambled or have been gambling and willing to fill in the questionnaire. In the data processing and reporting, 46 questionnaires were excluded due to incomplete completion and non-return.

### Measures

Before data collection started, two pilot tests were conducted and the relevance of the theme ‘gambling’ to the Ethiopian context was checked, next to the understandability of the items. The questionnaires were first constructed in English and then translated to Amharic and back translated to English by native Amharic speakers to ensure the quality of the translations. Socio-demographic questions were asked to illicit gender, age, grade levels, ethnicity, religious background, and familial information like, family type, family monthly income, and highest educational level in the household.

#### Problematic Gambling

We used two different standardized tools to assess the extent of adolescent problematic gambling in our sample. The first tool was the Diagnostic and Statistical Manual of Mental Health Fourth-Version Adapted for Juveniles (DSM-IV-J) checklist which was developed by Fisher and was taken from Rainone and Gallati (2007). The DSM-IV-J is a 12-item checklist which assesses nine dimensions of problematic gambling: progression and preoccupation, tolerance, withdrawal and loss of control, escape, chasing, lies and deception, illegal acts, family and academic disruptions, and financial bailout. Responses are given in “yes” or “no” format. Gamblers who respond “yes” to four or more of the items were classified as “probable problematic/pathological gamblers,” adolescents who responded 2–3 items positively were classified as at risk for severe problematic gambling, and those who scored 0–1 positive response were categorized as non-problematic or social gamblers (Derevensky and Gupta 2000; Rainone and Gallati 2007).

The second tool was the Gamblers Anonymous Twenty Questions (GA-20) questionnaire. Gamblers Anonymous (GA) is a fellowship of men and women who share their experiences, strength and hope with each other that they may solve their common problems and help each other to recover from gambling problems (retrieved from http://www.gansw.org.au/20%20questions.htm, & www.gamblersanonymous.org). The twenty items identify particular situations and behaviors that are typical of problematic/compulsive gamblers. The questions address the economic correlates of continued gambling, the psychological consequences of excessive gambling (e.g., difficulty in sleeping, remorse for excessive gambling, decreased ambition), and social correlates associated with excessive gambling behavior (e.g., difficult home life, arguments associated with gambling). An individual endorsing seven or more of the twenty items is considered to be a compulsive gambler while adolescents endorsing less or equal to six items are non-compulsive gamblers (Derevensky and Gupta 2000).

#### Correlates of Problematic Gambling

To identify risk factors of problematic gambling we assessed personal, social and environmental variables assumed to be associated with problematic gambling. The variables were identified from literature reviews. Personal risk factors (personal feelings) included measures of the extent to which gambling results in positive personal feelings, including self-esteem, reduction of negative feelings, and to cope with loneliness and distorted cognitions about the negative consequences of gambling, including false perception about winning huge amounts of money, and winning back previous losses. The social factors measured were for instance peer modeling of gambling, gambling to win peer acceptance, parental gambling behavior, loose parental regulation on gambling, and parents’ encouragement of gambling. Measures of environmental level factors included accessibility of gambling venues, for instance the accessibility of gambling on the pass ways of adolescents to their school, availability of gambling venues on the streets, and advertisement on gambling like winning huge money, winning cars, homes and etc. (see Table 1 for an overview of study variables, example items, scale measure and Cronbach’s alpha).

**Table 1:** Overview of study variables, example items, scale measure and Cronbach’s alpha

Measure and example items	Number of items	Sum scores	Cronbach’s alpha (*α)*
*Personal feelings (0* = *never, 1* = *rarely, 2* = *sometimes, 3* = *usually, 4* = *always)*	8	32	0.82
1. How often have you played games to enhance your self-confidence?			
2. How often have you played games to reduce tension?			
3. How often have you played games to forget arguments with your parents?			
*Social risk factors (peer and family influences (0* = *never, 1* = *rarely, 2* = *sometimes, 3* = *usually, 4* = *always)*	17	68	0.78
Peer influences (9-items)			
1. How often have you wagered or betted on sport events outcome to win peers acceptance?			
2. How often do you play games for money to get appreciation from your friends?			
Family influences (8-items)			
1. How often have you played games for money with your parents?			
2. How often your family member has takes you to the place where various games are played in money?			
*Environmental factors (availability and perceived media influences) (0* = *never, 1* = *rarely, 2* = *sometimes, 3* = *usually, 4* = *always*	16	64	0.81
Availability of gambling venues (8-items)			
1. How often have you spent holidays or weekends at game houses?			
2. How often have you visited game houses during the past years?			
*Perceived media influence (8*-*items)*			
1. To what extent has radio advertisement influenced your gambling behavior positively?			
2. To what extent has television advertisement influenced your gambling behavior positively?			
*Drug abuse (0* = *never, 1* = *rarely, 2* = *sometimes, 3* = *usually, 4* = *always)*	6	24	0.87
1. How often do you smoke hashish			
2. How often do you smoke cigarettes			
3. How often do you drink alcohol			
*Psychological impacts (0* = *never, 1* = *rarely, 2* = *sometimes, 3* = *usually, 4* = *always)*	7	28	0.82
1. How often do you feel depressed after you played games in money?			
2. How often do you feel nervous after you played games in money?			
3. How often do you feel that you have disturbing thoughts as a result of playing games in money?			
*Social impacts (0* = *never, 1* = *rarely, 2* = *sometimes, 3* = *usually, 4* = *always)*	14	46	0.87
1. How often have you get quarreled or fought with your friends on the game playing?			
2. How often have you got in conflict with your family members as results of you play games in money?			
3. To what extent do your relationship is becoming in problem with your friends due to you play games?			
*Economic impacts (0* = *never, 1* = *rarely, 2* = *sometimes, 3* = *usually, 4* = *always)*	6	24	0.82
1. After losing money on games, how often have you returned another day to try and win back money you lost?			
2. How often have suffered from losing all money you have?			
3. How often have you taken money from school lunch or taxi without permission to spend on game playing or betting?			
*Diagnostic and Statistical Manual for Mental Health Fourth Version, Adapted for Juveniles (DSM*-*IV*-*J) (0* = *no, 1* = *yes)*	12	12	0.74
1. Have you found yourself thinking about playing games in money or planning to play games in money (often)?			
1. Have you needed to play games with more and more money to get the amount of excitement that you wanted?			
2. Have you felt bad or fed up when you tried to cut down or stop playing games in money (sometimes or often)?			
*Gamblers anonymous twenty items (GA*-*20) (0* = *no, 1* = *yes)*	20	20	0.81
1. Did you ever lose time from work or school to playing games in money?			
2. Has playing games in money ever made your home life unhappy?			
3. Did your playing games in money affect your reputation			

## Results

### Socio-Demographic Characteristics of Participants

In the first report of this study (Abdi 2011), 307 out of 422 contacted students (about 73 %) reported that they had participated or are currently participating in gambling activities. Out of the 307, 46 responses were discarded due to incomplete filled-out questionnaires. For the current report, out of the 281 responses, 20 responses were missed in the process and the responses of 261 students of grade 9 (164 or 62.8 %) and grade 10 (97 or 37.2 %) were analyzed. The number of males in the current study was 146 (55.9 %) and females 115 (44.1 %). The ages of the adolescents ranged from 12 to 21 years with mode 17, mean 16.39, standard deviation 1.42, and median 16.30. Most of the students were from intact family (86.2 %) and only one student was institutionalized. Regarding the ethnic and religious background aspect of the students, most of the participants were from the Amhara and Oromo ethnic groups with Christianity and Islamic religions being the most reported religious backgrounds of the participants. See Table 2 for complete summary of the socio-demographic characteristics of the participants.

**Table 2:** Socio-demographic variables of the participants

Variable	Frequency (%)
Gender	
Male	146 (55.9)
Female	115 (44.1)
Age	
12–15	78 (29.9
16–18	161 (61.7)
19–21	22 (8.4)
Grade	
9	164 (62.8)
10	97 (37.2)
Ethnicity	
Amhara	104 (39.8)
Oromo	89 (34.1)
Tigire	31 (11.9)
Gurage	27 (10.3)
Other	10 (3.8)
Religion	
Orthodox	227 (87.0)
Protestant	16 (6.1)
Islam	15 (5.7)
Catholic	1 (0.4)
Other	2 (0.8)
Family type	
Intact	225 (86.2)
Separated/divorced	15 (5.7)
Remarried	4 (1.5)
Institutionalized	1 (0.4)
Lives with relatives	16 (6.1)
Family monthly income (in Ethiopian currency, ETB, 1US $ = 18.79)	
150–350	40 (15.3)
351–550	44 (16.9)
551–750	32 (12.3)
751–1,000	53 (20.3)
1,001–1,500	50 (19.2)
1,501–2,000	20 (7.7)
>2,000	22 (8.4)
Highest educational level	
Illiterate	47 (18.0)
1–8 grades	78 (29.9)
High school completed or grades 10/12 completed	90 (34.5)
College diploma	27 (10.3)
Degree and post-graduate courses	19 (7.3)

### Forms of Gambling Activities Among the Adolescents

**Fig. 1:**
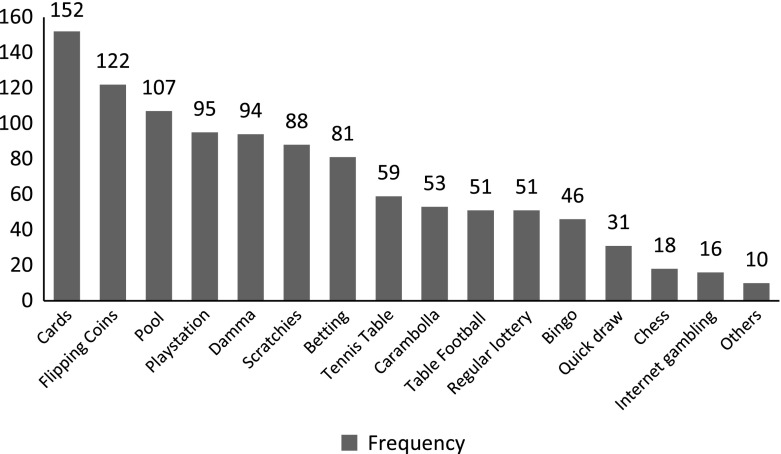
Forms of gambling activities among the adolescents

Adolescents engaged in various forms of gambling activities. The questionnaire identified 16 forms of local gambling activities. The bar graph presented in Fig. 1 reveals that the most frequently reported form of gambling activity is playing cards followed by flipping coins and pool gambling while Internet gambling is among the least reported forms.

### Risky Gambling Behavior

We used the DSM-IV-J and GA-20 tools to assess the extent to which adolescents were at risk for problematic gambling or were indeed problematic gamblers and non-compulsive or compulsive gamblers. Table 3 shows the distribution of respondents across the continuum of gambling behaviors (from social/non-problematic gambling to at risk for severe problematic gambling and problematic gambling) on the DSM-IV-J and non-compulsive to compulsive gamblers on the GA-20 tools.

**Table 3:** The distribution of respondents across the continuum of gambling categories of on DSM-IV-J and GA-20 for males and females

Tools	Category	Total (%)	Frequency (%)		Chi square
			Male	Female	
DSM-IV-J	Social gamblers	147 (56.3)	76 (61.7)	71 (52.0)	χ^2^ = 0.170, df. = 1, *p* = 0.68
	At risk for severe problematic gambling	96 (36.8)	58 (39.8)	38 (33.0)	χ^2^ = 4.167, df. = 1, *p* = 0.05*
GA-20	Probable problematic gambler	18 (6.9)	12 (8.2)	6 (5.2)	χ^2^ = 2.000, df. = 1, *p* = 0.16
	Non-compulsive gamblers	236 (90.4)	129 (88.4)	107 (93.0)	χ^2^ = 2.050, df. = 1, *p* = 0.15
	Compulsive gamblers	25 (9.6)	17 (11.6)	8 (7.0)	χ^2^ = 3.240, df. = 1, *p* = 0.07
	Total	261 (100)	146 (100)	115 (100)	

* Significant at *p* = 0.05

According to the DSM-IV-J checklist, the majority of our samples (56.3 %) were non-problematic or social gamblers, while 36.8 % were at risk for severe problematic gambling and 6.9 % were probable problematic/pathological. According to the GA-20 criteria, 90.4 % were reported to be non-compulsive gamblers while 9.6 % were compulsive gamblers.

Chi square analysis was conducted to reveal whether there is a significant difference in number of males and females on each level of gambling behaviors. The Chi square analysis revealed that males and females only significantly differ on the extent to which they are at risk for severe problematic gambling according to the DSM-IV-J checklist, with males being more at risk than females. The GA-20 checklist revealed that there is no significant gender difference to become compulsive gamblers. But the analysis revealed that males reported more compulsive gambling behaviors than females.

### Bivariate Correlation

The bivariate correlations between the study variables, including the univariate associations between the personal, social and environmental factors and the two measures of problematic gambling are presented (see Table 4).

**Table 4:** Bivariate correlations among the gambling variables

Measures	DSM-IV-J	GA-20	Personal feelings	Social factors	Environmental factors	Drug abuse	Psychological impacts	Social impacts	Economic impacts
DSM-IV-J	1								
GA-20	0.807^**^	1							
Personal feelings	190^**^	0.244^**^	1						
Social factors	0.254^**^	0.316^**^	0.455^**^	1					
Environmental factors	0.297^**^	0.331^**^	0.437^**^	0.549^**^	1				
Drug abuse	0.389^**^	0.393^**^	0.036	0.089	0.203^**^	1			
Psychological impacts	0.417^**^	0.476^**^	0.320^**^	0.439^**^	0.462^**^	0.218^**^	1		
Social impacts	0.506^**^	0.509^**^	0.290^**^	0.370^**^	0.469^**^	0.295^**^	0.554^**^	1	
Economic impacts	0.477^**^	0.514^**^	0.333^**^	0.383^**^	0.411^**^	0.341^**^	0.548^**^	0.699^**^	1

** Correlation is significant at the 0.01 level (2-tailed)

Adolescents’ scores on DSM-IV-J and GA-20were strongly and positively correlated. For the personal risk factors, positive associations with both measures of problematic gambling were found for personal feelings, drug abuse and perceived psychological, social and economic impacts. In addition, social risk factors and availability of gambling venues were positively correlated with both measures of problematic gambling.

Next, we employed regression analysis to explore the unique contribution of the univariate correlates in explaining problematic gambling for both dependent measures separately. For the measure of problematic gambling using the DSM-IV-J, the set of significant univariate correlates explained 34.9 % of the variance in problematic gambling with significant unique predictions for drug abuse, psychological impacts, and social impacts (see Table 5). For the GA-20, the model explained 39.1 % of the variance in problematic gambling showing unique contributions for again drug abuse, psychological impacts and social impacts, but also for economic impacts (see Table 6).

**Table 5:** Regression analysis of gambling variables on the DSM-IV-J

Variable	B	Std. error	Beta	t.	Sig
(Constant)	0.445	0.207		2.148	0.033*
Personal feelings	0.002	0.014	0.010	0.170	0.865
Social factors	0.005	0.011	0.027	0.407	0.684
Environmental factors	−0.002	0.012	−0.010	−0.150	0.881
Drug abuse	0.093	0.021	0.237	4.332	0.000**
Psychological impacts	0.045	0.021	0.141	2.117	0.035*
Social impacts	0.058	0.017	0.261	3.439	0.001**
Economic impacts	0.050	0.030	0.127	1.670	0.096

R^2^ = 0.349, F(7, 253) = 19.398, *p* < 0.01, ** = *p* < 0.01, * = *p* = 0.05

**Table 6:** Regression analysis of gambling variables on the GA-20

Variable	B	Std. error	Beta	t.	Sig
(Constant)	0.626	0.390		1.604	0.110
Personal feelings	0.018	0.026	0.040	0.693	0.489
Social factors	0.023	0.022	0.069	1.088	0.278
Environmental factors	−0.006	0.022	−0.018	−0.283	0.777
Drug abuse	0.180	0.040	0.236	4.454	0.000**
Psychological impacts	0.122	0.040	0.197	3.068	0.002**
Social impacts	0.082	0.032	0.188	2.560	0.011*
Economic impacts	0.125	0.056	0.163	2.204	0.028*

R^2^ = 0.391, F(7, 253) = 23.230, *p* < 0.01, ** = *p* < 0.01, * = *p* = 0.05

## Discussion

The study showed that personal factors/feelings (tension reduction, self-confidence, drug abuse, male gender), social factors (peer and family risk factors), and environmental factors (media advertisement and availability of gambling venues) contribute to problematic gambling among high school students in Addis Ababa, Ethiopia. International studies on problematic gambling reveal similar risk factors for problematic gambling, among which male gender, risk seeking tendencies, low self-esteem, depression and suicide ideation; social factors including peer influences and parental gambling; and environmental factors like advertisement on gambling have been found to be positively associated with problematic gambling continuum (e.g., Carlson and Moore 1998; Dane et al. 2004; Delfabbro et al. 2005; Lambos et al. 2007; Winters et al. 2002).

In addition, we explored various types of gambling activities adolescents engaged in. The research findings indicated that the most frequently played gambling forms among the high school students are playing cards, flipping coins, pool gambling and playstation, while Internet gambling is among the least reported. Delfabbro et al. (2005) in their study of adolescent gambling in Australian Capital Territory (ACT) of students of ages between 7 and 12 years revealed that private card games (39.8 %) and bingo/scratchies (40.5 %) were the most frequently reported gambling activities while betting on racing and sporting events were also popular (32 and 26 % respectively). In another study conducted among adolescents in Oregon (Carlson and Moore, 1998) purchasing raffle tickets (41 %) was the most frequently cited gambling activity, followed by betting on sports with friends or relatives (32 %); playing cards (31 %), and betting on games of skill, such as pool or bowling (25 %).

A game typical to Ethiopia is Carambolla, which ranked 9th among the identified 16 gambling activities and about 1 out of 5 gamblers play Carambolla. Carambolla is very closely similar with the Italian game of Bocette (http://billiardtraveler.blogspot.com/2011/10/billiards-in-hottest-inhabited-village.html). The variations in the prevalence of gambling activities may also be related to the differences in cultural backgrounds, age, legal and other social aspects of the adolescent gamblers.

The present research findings are of importance for future researches on problematic gambling in Ethiopia by serving as a benchmark an providing intervention targets for the evidence-based design of educational interventions to prevent problematic gambling (for a protocol to develop behavior change programs, see for example Bartholomew et al. 2011; Kok et al. 2004). However, one should be cautious in generalizing the findings to other populations as this study was the first study into risk factors for problematic gambling conducted in Addis Ababa, Ethiopia, which should be replicable in other settings and populations before generalizing. Also this study is conducted in the capital city of the country in which adolescent lifestyle is strongly influenced by Western culture, which in turn is very different from that of the countryside. Therefore, it is difficult to consider these findings as representative of all adolescents in the country. Finally, some participants after filling out the questionnaire handed it in with their teacher. This might have affected the reliability of their reporting, however we doubt whether this has affected our findings. Participants were not asked for any personal information to ensure anonymity. It would also have been difficult for the teachers to check each and every student’s response because almost about 73 % of the students contacted participated in the study. Furthermore, it is known that gambling in Ethiopia is technically not illegal and teachers have no legal ground to take disciplinary measures on the students.

To conclude, this study has contributed to the understanding of important variables of problematic gambling in Ethiopia. This is a pioneering work and provides a core list of possible intervention targets by identifying relevant correlates of problematic gambling. By doing so, it provides invaluable information for the systematic design and evaluation of evidence-based educational interventions to prevent problematic gambling in adolescents in Ethiopia.
